# Colour vision in nocturnal insects

**DOI:** 10.1098/rstb.2021.0285

**Published:** 2022-10-24

**Authors:** Eric Warrant, Hema Somanathan

**Affiliations:** ^1^ Department of Biology, Lund University, Sölvegatan 35, 22362 Lund, Sweden; ^2^ School of Biology, Indian Institute of Science Education and Research, Maruthamala PO, Vithura, Thiruvananthapuram, Kerala 695551, India

**Keywords:** insect, hawk moth, bee, nocturnal vision, colour vision, light pollution

## Abstract

The ability to see colour at night is known only from a handful of animals. First discovered in the elephant hawk moth *Deilephila elpenor*, nocturnal colour vision is now known from two other species of hawk moths, a single species of carpenter bee, a nocturnal gecko and two species of anurans. The reason for this rarity—particularly in vertebrates—is the immense challenge of achieving a sufficient visual signal-to-noise ratio to support colour discrimination in dim light. Although no less challenging for nocturnal insects, unique optical and neural adaptations permit reliable colour vision and colour constancy even in starlight. Using the well-studied *Deilephila elpenor*, we describe the visual light environment at night, the visual challenges that this environment imposes and the adaptations that have evolved to overcome them. We also explain the advantages of colour vision for nocturnal insects and its usefulness in discriminating night-opening flowers. Colour vision is probably widespread in nocturnal insects, particularly pollinators, where it is likely crucial for nocturnal pollination. This relatively poorly understood but vital ecosystem service is threatened from increasingly abundant and spectrally abnormal sources of anthropogenic light pollution, which can disrupt colour vision and thus the discrimination and pollination of flowers.

This article is part of the theme issue ‘Understanding colour vision: molecular, physiological, neuronal and behavioural studies in arthropods’.

## 1. Introduction

For a human observer, the dim nocturnal world is one that is black and white. The brilliant colours of objects that we so easily see in bright sunshine eventually fade to dim shades of grey as light levels fall at dusk [1]. During this transition from day to night, our three spectral classes of cone photoreceptors—whose responses to light are compared by the visual system to fill our world with colour—begin to fail, their increasingly weak signals becoming less and less reliable as light levels fall [2,3]. At the same time, our rod photoreceptors begin to shoulder the role of providing us with visual impressions, eventually being solely responsible. But unlike the cones, the rods—which in humans and nearly all other vertebrates come as a single spectral class—are incapable of supporting colour vision. With only a handful of known exceptions [4,5], the ability of terrestrial vertebrates to see colour is entirely restricted to daylight hours. Indeed, those vertebrates that have evolved a strictly nocturnal lifestyle (e.g. owl monkeys and bushbabies) have often lost one or more spectral classes of cones to instead invest in high-sensitivity monochromatic night vision [6–9].

But even though we ourselves, and essentially all other terrestrial vertebrates, are unable to see colour at night does not mean that the nocturnal world is colourless. Nor does it mean that colour cannot be seen by any animal at night. The physical colour of an object depends on only two things—the irradiance spectrum of natural daylight, and the spectral reflectance properties of the object's surface. Even though (as we will see) the irradiance spectrum can differ significantly as day transitions to night, the spectral reflectance properties of objects are invariant. In other words, the world is as equally colourful at night as it is during the day, and the advantages of colour vision—for recognizing food, mates, habitats or homes [3]—are equally great at night as they are during the day. Nonetheless, due to the problems of visual noise in very dim light, the discrimination of colour at night is far from trivial [3], and not surprisingly this ability is rare among animals. Among vertebrates, the only species known to discriminate colour at light levels dimmer than weak moonlight are the nocturnal helmet gecko *Tarentola chazaliae*, with its unusual all-cone retina [4], and two anurans, the toad *Bufo bufo* and the frog *Rana temporaria*, each with two spectral classes of rods that (incredibly) mediate spectral opponency and colour vision close to visual threshold [5].

**Figure 1.**
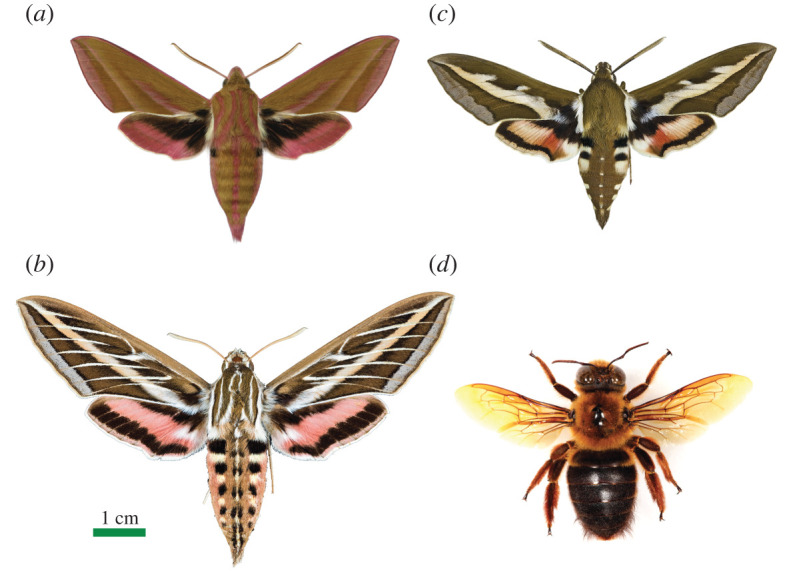
The four nocturnal insect species currently known to possess colour vision (as determined by behavioural experiments). (*a*) The elephant hawk moth *Deilephila elpenor* [10]. (*b*) The white-lined sphinx moth *Hyles lineata* [11]. (*c*) The bedstraw hawk moth *Hyles galii* [11]. (*d*) The carpenter bee *Xylocopa tranquebarica* [12]. The scale bar applies to all panels. Photo credits: (*a*) SLU Artdatabanken, Sweden (Karl Jilg); (*b,c*) Wikimedia Commons (Didier Descouens); (*d*) Nicolas Vereecken. (Online version in colour.)

Within the insects, nocturnal colour vision is known only from a small number of night-active pollinators, notably moths and bees (figure 1), where it is likely used to distinguish flowers [10,11]. The first evidence for nocturnal colour vision in any animal was obtained in the night-active elephant hawk moth *Deilephila elpenor* (figure 1*a,* [10]), and since then it has been identified in two other hawk moths (*Hyles lineata* and *Hyles gallii*, both active by day and night: figure 1*b,c* [11]), as well as in a large nocturnal carpenter bee (*Xylocopa tranquebarica*, figure 1*d* [12]). As for diurnal insects with colour vision, all of these insects have a number of spectral classes of photoreceptors—in their case a UV-, blue- and green-sensitive class—whose signals are compared to create colour vision (via opponent mechanisms at higher levels in the visual system). Two other nocturnal insects—the bull ant *Myrmecia vindex* [13] and the carpenter ant *Camponotus rufipes* [14]—also have three spectral classes of photoreceptors, and thus the potential for colour vision, but as yet this ability has not been demonstrated.

Thus, despite their small eyes and brains, some nocturnal insects have the capacity to distinguish colour at night, an ability that could well turn out to be more widespread among insects than among vertebrates. Indeed, the presence of at least three opsin classes in selected species from most superfamilies of nocturnal moths [15] strongly supports this notion. In this review we will first describe the world of colour at night and what limits reliable colour vision in dim light (and how this might be overcome). We will then turn our attention to the evidence for colour vision in nocturnal insects, and discuss the ecological roles it may have for nocturnal insect pollinators. Finally, we will consider the possible threats of light pollution on the reliability of colour vision at night and its possible effects on nocturnal pollination.

## 2. The colours of night

As we mentioned above, the physical ‘colour’ of an object—that is, the spectrum of light reflected from its surface—depends essentially on two things: (i) the spectrum of irradiance and (ii) the spectral reflectance properties of the object's surface (which remain constant). Exactly how an animal perceives this ‘colour’ depends on how the visual system is built [11]. The spectral transmission characteristics of the ocular media through which the light passes, the number of spectral classes of photoreceptors which sample the resulting spectrum of light incident on the retina (which can vary from as few as one class to well over ten), the absorption peak-wavelengths of each of the spectral classes and the manner in which signals from the different classes are neurally processed, all determine the object's actual ‘colour’ as perceived by an animal. In other words, an object illuminated by a certain spectrum of light (e.g. sunlight) will differ in colour for one animal species to the next.

But even for an individual animal, a change in irradiance spectrum will change the spectrum of light reflected from an object's surface, and thus potentially alter the perception of the object's colour. For normal natural variations in irradiance spectrum, such as the green-shifted spectrum experienced beneath a dense forest understorey [16] compared to the spectrum experienced under an open sky, the visual system is able to compensate for such variations to preserve the perceived colours of objects. These neural processes of compensation—collectively referred to as ‘colour constancy’ [17]—ensure that we see a red apple as red irrespective of whether we look at it in a forest or in an open field.

### (a) Natural and unnatural sources of illumination

**Figure 2.**
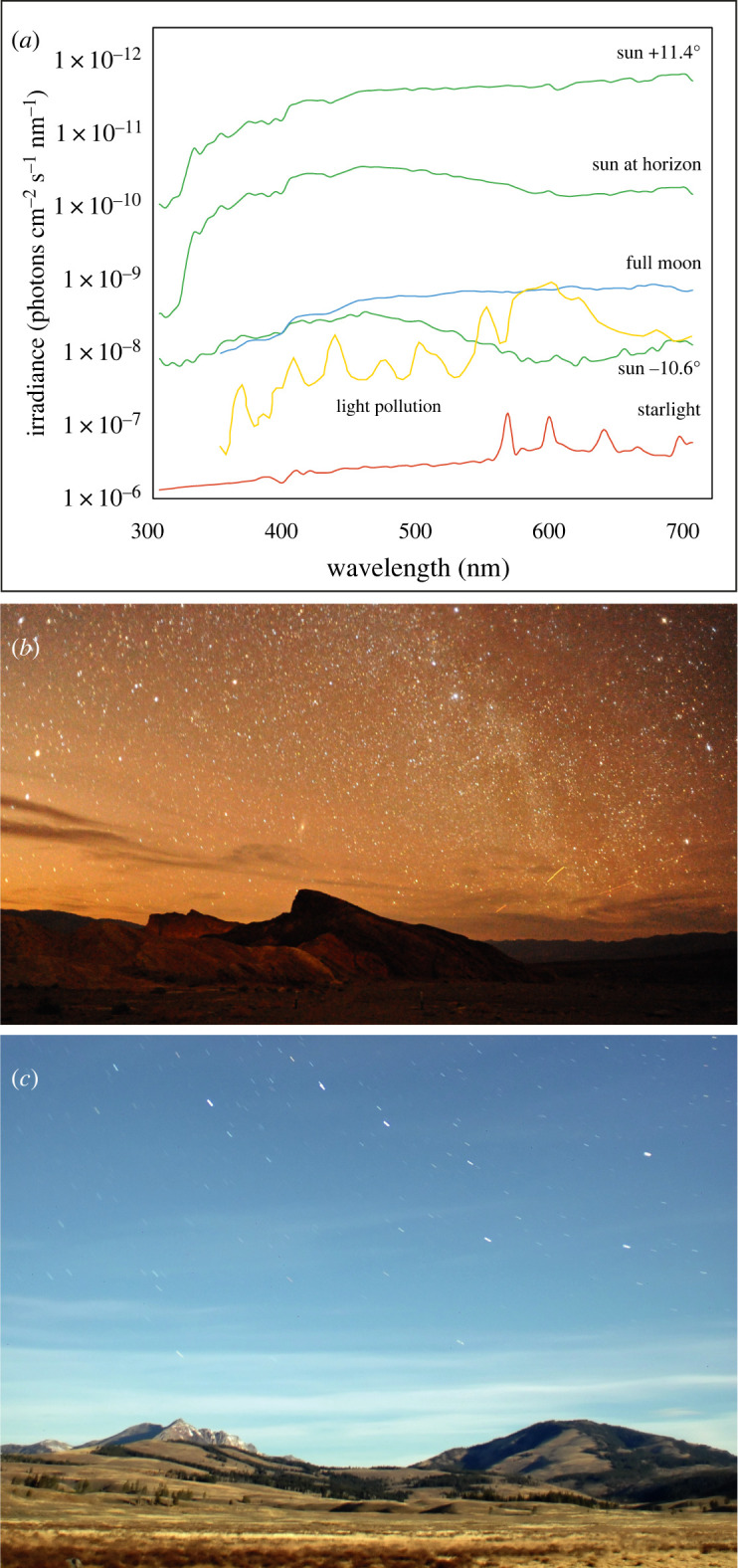
The spectral properties of light in terrestrial habitats. (*a*) The irradiance spectra of sunlight (green curves), moonlight (blue curve), starlight (red curve) and light pollution (yellow curve) in a terrestrial habitat (spectra were measured on a near-cloudless night). Sunlight spectra are shown just prior to sunset (sun elevation +11.4°), at sunset (sun at horizon) and just after sunset (sun elevation –10.6°). (*b*) A 62 s exposure taken on a moonless night in Death Valley National Park, California (Nikon D700, Nikon 20 mm f2.8 lens, f/2.8, ISO 6400). (*c*) A 148 s exposure taken three hours after sunset in the northwestern part of Yellowstone National Park (Nikon D70, Nikkor 20-mm lens, f/2.8, ISO 400). An almost full moon had recently risen on the eastern horizon. The scene appears as it would during the day (with the exception of the stars). Panel (*a*) adapted from Johnsen *et al.* [18]; panel (*c*) by Joseph Shaw, used with kind permission. Figure from Warrant & Johnsen [19]. (Online version in colour.)

There are three main sources of natural illumination on the Earth—the sun, the moon and the stars—and their spectra differ significantly (figure 2*a*, [18–20]).

The light experienced by a day-active (diurnal) terrestrial animal is completely dominated by direct and indirect light from the sun, a blackbody radiator whose broad-spectrum depends on its temperature (around 5800 K). Owing to the filtering effects of the ozone layer and other atmospheric constituents, this spectrum is narrowed by absorption in both the ultraviolet (UV) and the infrared before reaching the Earth's surface. The wide dome of the sky, while considerably dimmer (per unit area) than the sun, is also substantially bluer because of atmospheric (Rayleigh) scattering of the downwelling sunlight, which is more pronounced at shorter wavelengths. Since it is much larger than the disc of the sun, the blue sky contributes a significant fraction of the shorter wavelength light seen by diurnal animals (i.e. in the 300–500 nm range) and affects the final measured spectrum of skylight irradiance (figure 2*a*, [18]).

As the sun's elevation declines from a high angular value at midday to 0° at sunset, the daylight intensity at any one location on the Earth drops by approximately 100 times (figure 2*a*). By the time the sun has further sunk to 18° below the horizon (signalling the end of astronomical twilight and the onset of true night), light levels on a moonless night will have fallen a further 1–10 million times, although on a night lit by a full moon, light levels will be around 100 times brighter than this minimum. Cloud cover, or the presence of a dense forest canopy, can each further reduce light levels at any time of day by 10–100 times. Thus, from an open sunny meadow on a clear summer's day to the floor of a dense rainforest on a moonless and heavily overcast night, the light intensity difference could be up to 11 orders of magnitude [21,22], with a significantly greater proportion of this range occurring after sunset.

The transition from day to night (and night to day) brings a considerable change in the spectrum of daylight striking the surface of the Earth (figure 2*a*, [18]). As the sun drops close to the horizon, its light becomes dominated by longer wavelengths and it acquires the typical orange-red colour of sunset. But as the sun falls to just a few degrees below the horizon, sunlight is forced to travel a greater distance through the atmosphere. This makes it intensely blue (figure 2*a*), since longer wavelengths are filtered out by the intervening ozone. The blue twilight fades as the sun sets further.

On nights when the moon is absent, the sun's blue twilight is replaced by a dimmer and much redder light that comes from the stars, particularly from stars we cannot see—the vast numbers of red dwarfs emitting long wavelength light [23]. The stars we do see are much broader in spectrum (and thus appear white), but their contribution to the starlight irradiance spectrum is comparatively small. Airglow, which causes sharp peaks in the starlight spectrum (figure 2*a*), also contributes. The redder illumination of starlight can be readily seen in an image of Death Valley (California) obtained on a moonless night with an exposure of 62 s (figure 2*b*). The landscape is distinctly orange, although lacking colour vision at night we would fail to notice this.

By contrast, a landscape bathed in moonlight looks remarkably similar to the same landscape bathed in sunlight (figure 2*c*), a reflection of the fact that moonlight is simply reflected sunlight. The moon behaves as a near-perfect mirror that redirects the sun's light, although upon reflection the moon does absorb a portion of the UV and thus slightly alters the spectrum of the reflected sunlight (creating a weak red bias) [24].

Apart from these natural sources of illumination, the last century has witnessed a steady increase in illumination produced by humans. This illumination competes with, and in urban settings often overrides, the natural illumination, potentially causing significant problems for nocturnal animals that depend on vision for orientation and other ecological purposes [25,26]. Until recently, this ‘light pollution’ has mostly been generated by mercury bulbs and sodium lamps whose spectra are significantly red-biased (figure 2*a*). But these lamps are now being replaced by broad-spectrum white light-emitting diode (LED) lamps [27]. We are only now starting to understand how light pollution impacts the visual behaviour of nocturnal animals, and research on this topic is becoming increasingly prominent [e.g. 26,28,29]. We will return to the impacts of light pollution on colour vision and pollination at the end of this review.

### (b) The colours of ecologically relevant objects at night

**Figure 3.**
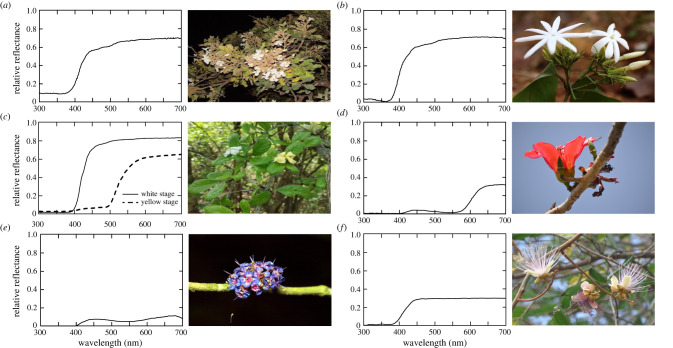
Nocturnal pollinators seldom feed from flowers that open exclusively at night. Night-blooming flowers tend to be white or creamish in colour. However, nocturnal insect pollinators are not restricted to visiting these colours. (*a*) White UV-absorbing flowers of *Heterophragma quadriloculare* have nocturnal anthesis and are pollinated by the nocturnal carpenter bee *Xylocopa tranquebarica.* (*b*) *Jasminum malabaricum* is a shrub with nocturnal anthesis and white UV-absorbing flowers that are visited by butterflies and bees during the day and by moths at night. (*c*) *Catenuregam spinosa* flowers open during the day and last several days changing colour from a UV-absorbing white to a bright yellow. Flowers are visited by honeybees and solitary bees during the day and by hawk moths and carpenter bees at night. (*d*) *Bombax ceiba* flowers are red and largely bird-pollinated during the day, but are visited by the nocturnal carpenter bee *X. tranquebarica* at night. (*e*) *Memecylon umbellatum* flowers are blue and open during the day when they are visited by honeybees and diurnal carpenter bees. They are visited by the nocturnal carpenter bee *X. tranquebarica* at night. (*f*) *Capparis zeylanica* flowers open at dusk and are visited at night by bats and are visited the following day by bees. Photo credits: (*a*) Hema Somanathan, (*b*) Shatarupa Ganguly, (*c*) Balamurali MG, (*d*) Ajith Ashokan, (*e*) Ullasa Kodandaramaiah, (*f*) Kavya Mohan. Spectral reflectances of flowers were measured by Balamurali MG (*a,b,c,d*), Hempel de Ibarra (*e*) and Kavya Mohan (*f*).

As pollinators, all four insect species that are known to have nocturnal colour vision (figure 1) visit a variety of flowers in their native habitats (figure 3, [30]). Some of these flowers open only at night (e.g. the flowers of the cambuci tree *Campomanesia phaea*, which are pollinated by nocturnal bees [31]), while others are open day and night and are pollinated by both diurnal and nocturnal insects (e.g. the lilac *Syringa vulgaris* and the honeysuckle *Lonicera caprifolium*). To a human observer, a large fraction of these flowers appear pale or white, often glossy, and with a high contrast against green foliage (e.g. the New Mexico evening primrose *Oenothera neomexicana* or the night phlox *Zaluzianskya capensis*), and interestingly these flowers typically lack reflection in the UV part of the spectrum ([32]; see figures 3 and 7*a*). Other nocturnally open flowers can be blue (e.g. the woodland phlox *Phlox divaricata*), purple (e.g. the lilac) or yellow (e.g. the common evening primrose *Oenothera biennis*). Common to almost all night-opening flowers is a strong and typically sweet aroma, underlining the fact that nocturnal pollinators are heavily reliant on olfaction as well as vision for identifying and feeding from flowers [33,34]. Indeed, some species of flowers release their perfume exclusively at night in order to enhance their attractiveness to nocturnal pollinators (e.g. the honeysuckle).

Whether nocturnal insects can use body coloration for sexual signalling is unknown, although it has been implicated (but not proven) in one species of moth—the dot-underwing moth *Eudocima materna* [35]. Many nocturnal moths possess distinctive colour markings, including hawk moths, where the hindwings are often colourful (figure 1). However, compared to diurnal butterflies (that often use body coloration as a sexual signal), nocturnal moths are rather drably coloured, possibly for camouflage while resting during the day (e.g. [36]).

Of course, nocturnal insects may use colour vision in other contexts apart from seeking out flowers. As we mentioned earlier, colour vision is used for many ecological tasks, such as recognizing food, mates, rivals, predators, habitats or homes [3], and these uses are well known in day-active animals [37]. And since, as we have also said earlier, the nocturnal world is as equally colourful as the diurnal world, there is no reason why nocturnal animals with sufficiently sensitive eyes should not be able to use colour vision for the same purposes. Indeed, the nocturnal carpenter bee *Xylocopa tranquebarica* (figure 1*d*) could be trained to associate a colour with its nest entrance, suggesting a possible role for colour during homing [12]. But for nocturnal insects, this is so far the only demonstration of colour vision in a context other than flower visitation.

## 3. The difficulties of having colour vision at night

### (a) The problems

To see well at night, irrespective of whether vision is chromatic or achromatic, is far from trivial [3,22,38]. Even though the colours and contrasts of the visual world are essentially the same at night as they are during the day, the extreme paucity of light—which can be up to 100 billion times dimmer than sunshine—makes discrimination of these essential visual features highly unreliable. A large part of the problem lies in a severely diminished visual signal—eyes are forced to distinguish features of the world with vanishingly few photons. Compounding this is a second problem, visual noise. Part of this noise arises from the stochastic nature of photon arrival and absorption (which is governed by Poisson statistics). A photoreceptor that absorbs *N* photons during one visual integration time, will experience an uncertainty—or ‘photon shot noise’—of √*N* photons associated with this sample, that is, *N* ± √*N* photons [22,38–44]. This photon shot noise reduces the reliability of intensity discrimination and thereby the ability of the eye to distinguish contrast details in a scene. The signal-to-noise ratio (SNR), simply *N*/√*N*, or √*N*, improves with increasing photon catch, implying that photon shot noise, and contrast discrimination, is relatively worse at lower light levels. This famous ‘de Vries-Rose’ or ‘square root law’ of visual detection at low light levels indicates that the visual SNR, and thus contrast discrimination, improves as the square-root of photon catch.

Unfortunately, this is not the only source of noise. There are two further sources that also degrade visual discrimination by photoreceptors in dim light. The first of these, referred to as ‘transducer noise’, arises because photoreceptors are incapable of producing an identical electrical response, of fixed amplitude, latency and duration, to each (identical) photon of absorbed light. This source of noise, originating in the biochemical processes leading to signal amplification, degrades the reliability of vision [45–47].

The second source of noise, referred to as ‘dark noise’, arises because the biochemical pathways responsible for transduction are occasionally activated—even in perfect darkness [48]. This dark noise manifests itself in two ways: (i) a continuous low-amplitude fluctuation in measured electrical activity (sometimes called membrane or channel noise) and (ii) discrete ‘dark events’, electrical responses that are indistinguishable from those produced by real photons. The continuous component arises from spontaneous thermal activation of rhodopsin molecules or of intermediate components in the phototransduction chain (such as phosphodiesterase: [49]). The amplitude of this membrane noise is negligible in insects [45], but can be quite significant in vertebrate photoreceptors, particularly cones. ‘Dark events’ arise due to spontaneous thermal activations of rhodopsin molecules. These are rare in insects [45,47,50,51] but can occur with much higher regularity in vertebrate cones [52,53]. At very low light levels both components of dark noise can significantly contaminate visual signals [54], and even set the ultimate limit to visual sensitivity [55,56].

The problems of noise in dim light compound for colour discrimination. Since colour vision is based on opponent interactions between two or more spectral classes of photoreceptors, the ability of an eye to discriminate colour will be limited by the noise levels present in each class [57–59]. In particular, the number of colours that are reliably discriminated is limited by the product of the noise in each class—the greater the noise, the larger the differences in colour need to be before they can be discriminated and the smaller the total number of colours that are visible [60]. Thus, at dimmer light levels and/or higher noise levels, fewer colours can be seen. This is likely the reason why nocturnal colour vision is rare in vertebrates [3]. Because the photoreceptors of vertebrates are generally much noisier than those of insects, monochromatic vision tends to be the default at night [60].

### (b) The solutions

Since the problem for discriminating colour in dim light is a low visual SNR, the solution lies in optical and neural strategies that increase it, either by boosting the signal (e.g. by increasing the photon catch) or by reducing the noise (e.g. by averaging it out)—or ideally both. Insects have evolved many such strategies, and these are key to their remarkable visual abilities at night (for full reviews, see [44,61]).

**Figure 4.**
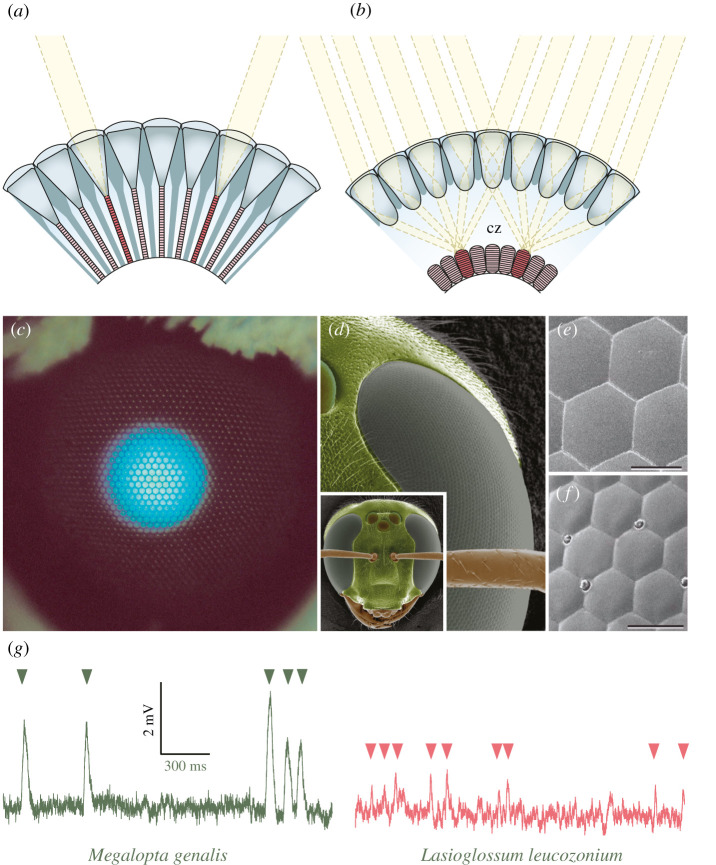
Optical and retinal solutions for colour vision in dim light. (*a*) Apposition compound eyes. Photoreceptors (pink) within each ommatidium (nine are seen here in cross-section) receive light exclusively from the single facet lens of their own ommatidium (since each ommatidium is sleeved by a layer of light-absorbing pigment granules that prevent light arriving from neighbouring ommatidia). This eye type is typically (but not exclusively) possessed by diurnal insects active in bright light. (*b*) Refracting superposition compound eyes. Here the lenses and photoreceptors are separated by a wide optically clear (i.e. pigment free) region known as the clear zone (cz). Owing to powerful radial gradients of refractive index, the lenses allow light from many hundreds of facet lenses to be focused onto single photoreceptors in the retina, thus dramatically increasing light capture. This eye type is typically (but not exclusively) possessed by nocturnal insects active in dim light. Figures courtesy of Dan-Eric Nilsson. (*c*) The superposition aperture of an unidentified moth revealed by unabsorbed incident axial light reflected from a tapetal mirror lining the back of the retina. (*d*) The nocturnal sweat bee *Megalopta genalis* has apposition compound eyes and three sensitive eyelets—the ocelli—located between the eyes. Even though it is likely that the eyes possess three spectral classes of photoreceptors, it has not as yet been demonstrated. Only a single class of photoreceptor (with peak sensitivity in the blue-green at 500 nm) has been demonstrated in the ocelli [64]. (*e,f*) The facet lenses of *M. genalis* are larger (*e*) than those of its day-active relative *Lasioglossum leucozonium* (*f*), and thus focus more light on the photoreceptors. Scale bars: 25 µm. (*g*) The responses of photoreceptors to single photons of light (arrowheads) are roughly five times larger in nocturnal (*M. genalis*) than in diurnal (*L. leucozonium*) sweat bees, indicating that the nocturnal photoreceptors have a higher gain (or amplification) in order to improve the reliability of vision in dim light. Adapted and redrawn from Frederiksen *et al.* [65]. (Online version in colour.)

Optically, signal amplitude can be improved by having an eye design that captures more light. In insects, this is achieved via the flexible design of their compound eyes. These eyes are constructed of (typically) thousands of tightly packed optical units known as ‘ommatidia’, thin cylindrical structures that each house a set of lenses that focus light from a single ‘pixel’ of the outside world onto a rod-like bundle of light-sensitive elements (called the ‘rhabdom’) provided by the photoreceptors directly below (for a complete description, see [62,63]). Externally, the packing array of ommatidia is marked by a crystalline matrix of hexagonal ‘facets’ (figure 4*d–f*), each being the curved external surface of the single corneal lens that supplies light to underlying rhabdom.

Compound eyes fall into two broad subtypes: apposition eyes (figure 4*a*) and superposition eyes (figure 4*b*). The essential difference between them lies in the number of facets that provide light to a single rhabdom. In apposition eyes, only one is involved. Light rays entering a corneal facet lens (which provides a pupil only a few tens of micrometres wide) are focused exclusively onto the rhabdom within the same ommatidium. In superposition eyes, many facets are involved: a single rhabdom instead receives light rays that enter a large number of corneal lenses (usually several hundred that form a wide pupil-like ‘superposition aperture’: figure 4*c*). And herein lies the optical advantage of superposition eyes for vision in dim light—the light signal on each rhabdom is boosted several hundred times. Not surprisingly, nocturnal insects very typically have this type of compound eye, including many beetles and most moths, such as our three colour-seeing hawkmoths in figure 1.

Remarkably though, despite their distinctly lower sensitivity, apposition eyes are found in all nocturnal ants, wasps and bees, including the giant Indian carpenter bee *Xylocopa* (figure 1*d*), and all of them are known to have extraordinary visual capacities in dim light [44,66]. As part of their response to a life at night, the eyes of these insects have evolved much larger facets (figure 4*e*) and significantly wider rhabdoms than found in their similarly sized day-active relatives (figure 4*f*), boosting their photon catch by about 30 times [66]. Obviously, a boost by 30 times, or even hundreds of times (as with superposition optics), does provide a great improvement in visual SNR, but on its own it is woefully insufficient to bridge the billion-fold difference in light levels typical from day to night. How is this shortfall met?

The answer (at least partially) lies in several impressive neural adaptations that further improve the visual SNR in dim light. Firstly, the photoreceptors of nocturnal insect eyes tend to be much slower and have significantly larger single-photon responses (i.e. higher transduction gain) than those of diurnal insects (figure 4*g*, [65,67–69]), adaptations that significantly improve the reliability of visual information in dim light [69,70].

Secondly, nocturnal insect visual systems possess peripheral neural mechanisms that sum photons of light in time and space [44,71]. Summation in time is somewhat analogous to having an increasingly longer exposure time as light levels fall—visual reliability can be improved by responding more slowly and building up a brighter image. But this only comes at a price: the resolution of events occurring rapidly in time, such as the passage of a fast-moving object, can be significantly degraded, potentially disastrous for a fast-flying nocturnal animal that needs to negotiate obstacles. Not surprisingly, substantial temporal summation is more likely to be employed by slowly moving animals.

**Figure 5.**
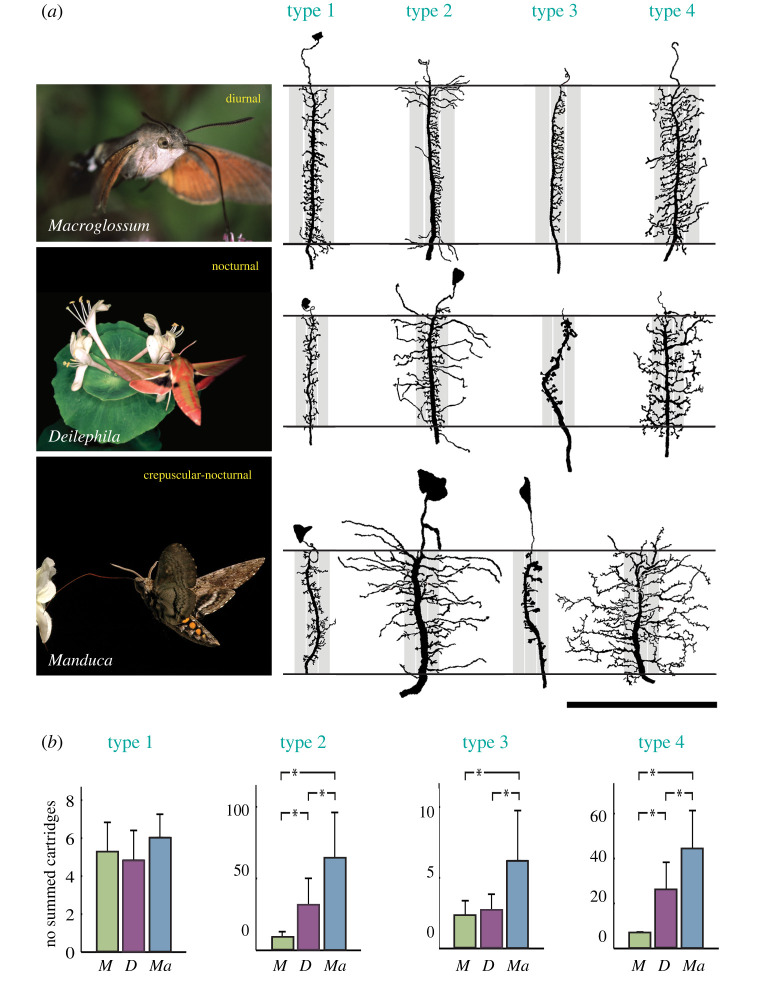
Spatial summation. (*a*) The morphologies of the four types of lamina monopolar cells (LMCs) found in hawkmoths active at different light levels: the diurnal hummingbird hawkmoth *Macroglossum stellatarum*, the crepuscular/nocturnal tobacco hornworm moth *Manduca sexta* and the strictly nocturnal elephant hawkmoth *Deilephila elpenor*. Note how the lateral dendritic branches of LMC types 2, 3 and 4 are much more extensive in the crepuscular and nocturnal species than in the diurnal species. The grey vertical bars represent lamina cartridges (the processing unit corresponding to a single ommatidium)—in this case the ‘home’ cartridge of the LMC and two neighbouring cartridges, one on either side. Scale bar = 100 µm. (*b*) The number of neighbouring lamina cartridges reached by the lateral dendritic branches of type 1–4 LMCs in *Macroglossum* (*M*), *Deilephila* (*D*) and *Manduca* (*Ma*). Error bars show standard deviations and asterisks significant (*p* < 0.05) differences between species. Both panels adapted from Stöckl *et al.* [72]. (Online version in colour.)

Summation in space relies on activation of specialized laterally spreading neurons which couple visual channels (e.g. those arising in individual ommatidia) together into groups. Thus, instead of each channel collecting photons in isolation from a single small ‘pixel’ of the visual scene (as in bright light), the transition to dim light would generate summed groups of channels that each collect a much greater number of photons over a considerably wider visual angle, that is, from a considerably larger (and thus brighter) ‘pixel’. The neurons that mediate spatial summation in the visual systems of nocturnal bees and hawk moths turn out to be specialized highly branched lamina monopolar cells (LMCs) in the first optic neuropil of the brain (figure 5*a*, [72–75]), and these are capable of connecting large numbers of ommatidial visual channels together (figure 5*b*). However, just as for temporal summation, this strategy only comes at a cost: a simultaneous and unavoidable loss of spatial resolution. Despite being much brighter, the image becomes necessarily coarser.

Nonetheless, despite their negative consequences for spatial and temporal resolution, these summation strategies dramatically improve the visual SNR in dim light by enhancing the visibility of the coarser and slower features of the world at the expense of the finer and faster features. In the absence of summation nothing at all would be seen [67]. Good evidence for the presence of spatial and temporal summation has been found in the motion vision pathways of the nocturnal hawkmoth *Deilephila*, where they maximize the visibility of visual contrasts over four decades of light intensity and allow these moths to see at light intensities 100 times dimmer than otherwise would have been possible [76,77]. To preserve colour vision in dim light, spatial summation would need to occur separately for each spectral channel. As we will see below, this indeed likely happens in the visual system of *Deilephila*.

## 4. Evidence for colour vision in nocturnal insects

The most direct and convincing method to demonstrate the presence of colour vision in an animal is to use behavioural experiments. This method involves training an animal to associate a food reward with a coloured target, and afterwards testing the ability of a hungry animal to seek out this coloured target (now lacking food) within an array of identically sized grey targets that are lighter, identical or darker in shade than the learned colour. The variation in grey shade ensures that the animal truly has learned the colour of the target, and not simply its brightness, to identify and select the target in the test. These simple but elegant methods—first developed by Karl von Frisch over a century ago to demonstrate colour vision in honeybees [78]—were used to establish nocturnal colour vision in the four species of insects shown in figure 1 [10–12].

### (a) Colour vision in the elephant hawk moth *Deilephila elpenor*

**Figure 6.**
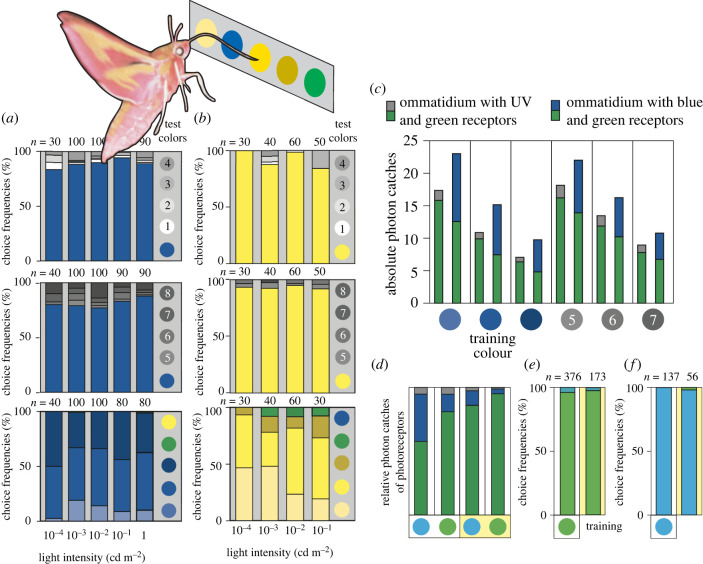
The behavioural proof for nocturnal colour vision and colour constancy in the elephant hawk moth *Deilephila elpenor*. (*a,b*) Test choice frequencies of hungry moths after being trained to associate food with a blue (*a*) or yellow (*b*) circular target. The learned target was presented together with (i) four grey targets having a shade that was either lighter (shades 1–4, upper row) or darker (shades 5–8, middle row) than the learned target, or (ii) targets having a lighter and darker shade of the learned colour, together with two targets having other colours entirely (lower row). Each stacked bar diagram gives the choice frequencies to each test colour (indicated by the coloured regions of each bar) at a specific light intensity (see abscissa) with the number of choices *n* given above each bar. (*c*) Absolute photon catches of all photoreceptors in the two types of ommatidia possessed by *Deilephila* (one type with two UV and seven green receptors, one type with two blue and seven green receptors) viewing six of the test colours (including the blue training colour) in starlight (10^−4^ cd m^−2^). In each stacked bar, the grey regions represent UV receptors, blue regions represent blue receptors, and green regions represent green receptors. (*d–f*) Colour constancy. Relative photon catches (*d*) in the three photoreceptor channels generated by turquoise and green targets illuminated by white (two left columns) or yellow (two right columns) illumination. Stacked bar coloured regions follow the conventions of (*c*). Moths trained to green targets (*e*) and turquoise targets (*f*) chose correctly in tests under two different illuminations, ‘white’ (left column) and ‘yellow’ (right column). Adapted from Kelber *et al.* [10]. (Online version in colour.)

**Figure 7.**
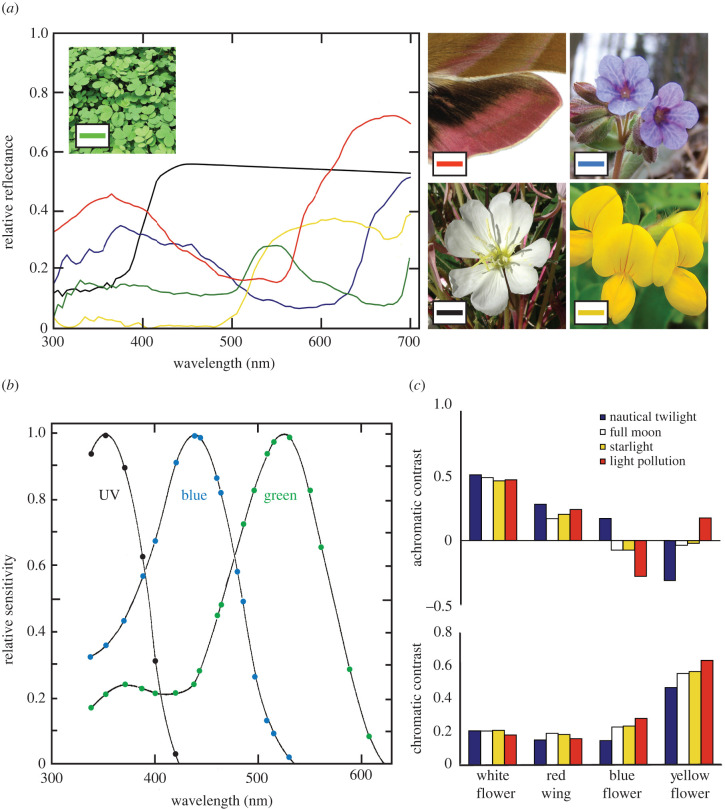
Modelling colour perception in the elephant hawkmoth *Deilephila elpenor*. (*a*) Reflectance spectra from natural objects that may have ecological relevance for hawk moths. A typical white flower (the New Mexico evening primrose *Oenothera neomexicana*, black curve [33]), a typical blue flower (the unspotted lungwort *Pulmonaria obscura*, blue curve [81]), a typical yellow flower (the birdsfoot trefoil *Lotus corniculatus*, yellow curve [81]) and the pink hindwing of the elephant hawk moth (red curve). Also shown is the reflectance spectrum of green foliage against which flowers are normally contrasted (green curve). Photo credits: Wikimedia Commons (photographers Stan Shebs, Marianne Cornelius-Kuyt and Tanukusreeharsha) and SLU Artdatabanken, Sweden (photographer Karl Jilg). Reflectance curves adapted from Johnsen *et al.* [18]. (*b*) The absorption spectra of the three different photoreceptor classes identified in *Deilephila*, with absorption peak wavelengths at 350 nm (UV-sensitive class), 450 nm (blue-sensitive class) and 525 nm (green-sensitive class). Adapted from Höglund *et al.* [79]. (*c*) Achromatic (upper) and chromatic (lower) contrasts of flowers and a hindwing seen against a green foliage background (whose spectral reflectances are shown in (*a*)) under four different irradiance spectra—twilight, moonlight, starlight and light pollution (from figure 2*a*). These contrasts are based on calculations of photons absorbed by each photoreceptor class, which depend on the irradiance spectrum of light that initially strikes the object (twilight, moonlight or starlight: figure 2*a*), the spectrum of light that the object's surface preferentially reflects (determined by the object's reflectance spectrum (*a*)), and the absorption spectrum of the photoreceptor itself (*b*). Adapted from Johnsen *et al.* [18]. (Online version in colour.)

The first demonstration of nocturnal colour vision in any animal was made in the elephant hawkmoth *D. elpenor* ([10]; figures 1*a* and 6). Serendipitously, in the early 1970s, Kurt Hamdorf and his group at the University of Bochum in Germany chose *Deilephila* as a model animal to study visual pigments. Their work led to the discovery of three spectral classes of photoreceptors in the retina, with absorption peaks in the UV, blue and green regions of the spectrum ([79,80]; figure 7*b*). With three classes of photoreceptors, the elephant hawkmoth was thus predicted to have trichromatic colour vision at night [82]. Almost a quarter of a century later, this prediction was proven to be correct [10].

To show the presence of colour vision in *Deilephila*, two training colours were used—blue (figure 6*a*) and yellow (figure 6*b*). In tests for colour vision, these learned circular coloured targets were presented together with circular grey targets, with shades that were both lighter (shades 1–4 in figure 6*a,b* upper rows) and darker (shades 5–8 in figure 6*a,b* middle rows) than the coloured target itself. In a further test, the learnt colour, together with lighter and darker shades of the same colour, were presented with two other colours—yellow and green when the learnt colour was blue (figure 6*a*, lower row), and green and blue when the learnt colour was yellow (figure 6*b*, lower row). The ability of *Deilephila* to choose the learnt colour in these test circumstances was determined at a number of luminance levels ranging from 1 cd m^−2^ (the luminance of a leaf-littered substrate under a clear sky 20–30 min after sunset, [22]) to 10^−4^ cd m^−2^ (the luminance of the same substrate under a clear sky in dimmest starlight).

At all light levels—including the dimmest starlight level—the learnt coloured target was the first target investigated by *Deilephila* (with its unfurled proboscis) in at least approximately 80% of trials when the coloured target was presented among grey targets (figure 6*a,b* upper and middle rows). This high level of performance was similar at all light intensities tested, proving that *Deilephila* not only has colour vision, but does so even in starlight. A human observer subjected to the same test begins to fail seeing colours at light levels around 100 times brighter. Even though *Deilephila* had difficulty distinguishing different shades of the learned colour, they rarely confused this colour with either of the other two colours presented simultaneously, even in starlight (figure 6*a,b*, lower rows). And incredibly, despite having apposition eyes, similar results were obtained in the nocturnal carpenter bee *Xylocopa tranquebarica* [12]. These bees are able to use colour cues in the context of homing.

In a further experiment—to test for the role of the UV receptor in colour vision—Kelber *et al*. [10] discovered that *Deilephila* could discriminate between two white targets, one that absorbed UV light and one that reflected it. Even though these two white targets looked identical to a human observer, the moth exclusively chose the learned colour (in this case the UV-absorbing target at a luminance of 0.01 cd m^−2^). This result strongly supports the idea that *Deilphila* possesses trichromatic colour vision (i.e. colour vision involving all three spectral classes of photoreceptors). The fact that other hawkmoths studied also have three photoreceptor classes (e.g. [83]) suggest that nocturnal trichromatic colour vision is likely to be widespread within this group of insects.

Remarkably, the ability of *Deilephila* to discriminate colour in starlight occurs when its UV-, blue- and green-sensitive photoreceptors are absorbing extremely low numbers of photons (figure 6*c*)—between 1 and 16 photons per visual integration time for the brighter blue and grey targets, and even fewer than this for the darker targets. As we discussed earlier, because the arrival of photons is random, the SNR is the square root of the photon catch—between 1 and 4 for *Deilephila* in starlight. Such low values would certainly be insufficient for *Deilephila* to discriminate colours in starlight. Indeed, the photon catches in each of the three spectral classes of photoreceptors resulting from the training colour and grey shade 6 are very similar (figure 6*c*), meaning that *Deilephila* should not be able to tell these two targets apart. But *Deilephila* clearly *can* tell them apart (figure 6*a*), implying that spatial and/or temporal summation is being used to boost the SNR and allow colour vision in starlight (as has been shown for motion vision [76]).

Since many species of hawk moths are crepuscular, they experience significant fluctuations in irradiance spectrum as the sun rises and sets (figure 2*a*). Such substantial changes in the illumination, and the accompanying changes in the spectrum of light reflected from objects in the visual scene, can potentially alter an animal's perception of object colour. However, as we mentioned earlier, this can be ameliorated by neural mechanisms that stabilize colour perception under a variable irradiance spectrum, a phenomenon known as ‘colour constancy’ [17], a hallmark of advanced colour vision systems. Not surprisingly, *Deilephila* reveals colour constancy (figure 6*d–f*, [10]). After learning to associate food with either a green or turquoise target under broad-spectrum white illumination (produced from a high-pressure mercury lamp), *Deilephila* had no problem distinguishing the rewarded colour from the unrewarded colour either under the white illumination or under a yellow-shifted illumination (created by introducing a coloured filter that removed light of wavelength below 450 nm: figure 6*e,f*). This is despite the fact that the relative photon catches of the three spectral classes of photoreceptors were very different under the two illuminations (figure 6*d*), indicating that these differences did not affect final colour perception.

### (b) Why does the elephant hawk moth have nocturnal colour vision?

As we mentioned earlier, many nocturnal mammals, like owl monkeys, have dispensed with colour vision altogether and instead rely on highly sensitive monochromatic vision to perform their nightly tasks. However, for a pollinating insect like *Deilephila*, the ability to distinguish coloured flowers might provide an obvious advantage for colour vision. But as we have seen, many (although not all) night-opening flowers are pale and bright (figure 3), apparently to maximize achromatic contrast cues in order to be detected. Indeed, *Deilephila* even tends to prefer such flowers [18]. So why have colour vision? Are there any advantages of nocturnal colour vision other than the obvious advantage for finding coloured flowers?

It turns out that the answer to this last question is ‘yes’. Under the highly variable irradiance spectrum encountered by a hawk moth like *Deilephila*, active from sunset to deep night (figure 2), object contrast is more reliably encoded by colour vision than by monochromatic vision [11]. This can be seen by modelling *Deilephila's* colour perception when confronted with different ecologically relevant objects under the varying natural irradiance spectra it normally encounters (figure 7; [18]). More specifically, this involves calculating the number of photons absorbed by each of the three different spectral classes of photoreceptors (figure 7*b*) when ecologically relevant objects—in this case three species of flowers (white, yellow and blue) and *Deilephila's* pink hindwing (figure 7*a*)—are seen contrasted against a green foliage background in twilight, moonlight and starlight (figure 2*a*). Together with accepted models of colour vision, these photon catches can be used to calculate the chromatic and achromatic contrasts of objects seen by *Deilephila* (figure 7*c*; [18]). What is immediately apparent is that achromatic contrast can vary wildly with irradiance spectrum (figure 7*c*), particularly for yellow and blue flowers where contrast can even change polarity (i.e. appear darker than the background under one illumination and brighter under another!). This is not the case for chromatic contrast which is significantly more stable. With the addition of colour constancy this stability turns out to be greater still [18].

Thus, colour vision—particularly with colour constancy—is likely to be far more reliable than monochromatic vision for viewing a wide variety of coloured objects under the variable illumination that *Deilephila* encounters from sunset to deep night. Interestingly though, for the bright pale flowers favoured by this moth, colour vision and monochromatic vision are similarly reliable, both revealing little variation in contrast under different illuminations (figure 7*c*). But for a nocturnal pollinator that prefers yellow and blue flowers, colour vision may represent a distinct advantage.

## 5. The role of colour vision in nocturnal pollination

### (a) Nocturnal pollinators

Similar to their diurnal counterparts, nocturnal insect pollinators visit flowers in search of food rewards, mostly nectar and pollen, but also for mating opportunities and suitable brood rearing sites. Thus, the nocturnal niche has potential benefits for both the pollinator and the flower. However, this partnership between night-blooming flowers and nocturnal pollinators is grossly understudied. Nocturnal pollinators visit flowers (during dusk) after or (during dawn) before diurnal pollinators, thus augmenting the quantity or the efficiency of pollination services rendered to plants (e.g. nocturnal hawk moths and noctuid moths on the white campion *Silene alba* [84]). Nocturnal hawk moths are often secondary pollinators of plant species where bats are the primary pollinators [85]. A few of these nocturnal partnerships can also be obligate and specialized, with the plant relying solely on one or very few pollinators, and vice versa (e.g. the senita cactus and senita moth [86]). The nocturnal environment is relatively safer and less competitive for pollinators, and the roles of predation and competition have been proposed as competing hypotheses for the evolution of nocturnality in pollinators [87,88].

Moths and beetles are the dominant invertebrate nocturnal pollinators [29,89], however, bees that are either facultatively or obligately nocturnal also pollinate flowers during crepuscular, matinal or nocturnal time periods. For example, the carpenter bee *Xylocopa tranquebarica* forages even during moonless nights in the Asian tropics [90] and neotropical sweat bees of the genus *Megalopta* are crepuscular pollinators although under the rainforest canopy light levels are extremely low at this time [91]. However, facultative rather than obligate nocturnality is more widespread among bee families [88]. For example, the Asian giant honeybee *Apis dorsata* [92] can forage at halfmoon light levels or brighter. Among vertebrates, bats are important nocturnal pollinators [93,94], while marsupials, lemurs, shrews and rodents are also known to pollinate flowers at night [95–97].

### (b) Colour and flower visitation by nocturnal pollinators

There are few detailed studies on nocturnal pollination by insects and most of these have focused on moths (e.g. [98,99]). Given the paucity of studies, we highlight some open questions regarding the nature of this partnership. Flowers present complex multimodal stimuli that are advertised to attract diverse pollinators by activating their sensory systems. Colour is a floral trait that is employed extensively by diurnal pollinators—including bees, butterflies, moths and hoverflies—to find flowers. Field observations and psychophysical behavioural experiments have established robust colour learning in diverse groups of diurnal pollinators. However, what role does floral colour play in the flower choices of nocturnal insect pollinators? In a recent review encompassing over a decade of published studies on pollination syndromes, Dellinger [100] found that colour was less reliable in predicting the identity of pollinator functional groups when compared to other floral traits such as reward or size. Notwithstanding this, colour is a salient cue that is learnt and recalled by nocturnal insect pollinators. As we have seen earlier, the nocturnal hawk moth *D. elpenor* can learn to associate the colour of artificial flowers with food rewards [10,11] and the nocturnal carpenter bee *X. tranquebarica* can learn to associate its nest entrance with a coloured landmark [12], even in starlight. However, how much (or how little) they rely on colour relative to other floral traits (e.g. scent) while searching for, and selecting, real flowers is still not fully understood.

A second question that remains largely unanswered is whether nocturnal insect pollinators are specialists on night-opening flowers. To examine this, we must briefly consider the discussion around floral traits and pollination syndromes (for a detailed discussion, refer to the extensive reviews [100–102] on this topic). Historically, plant–pollinator partnerships have been categorized into syndromes [103,104] which is articulated as the convergent evolution of floral traits by adaptation to the most efficient functional pollinator group. The concept of syndromes later drew much controversy and is still an unsettled debate. Several studies have found empirical support for syndromes, while several others did not. A recent meta-analysis of 417 plant species found overall support for pollination syndromes [105]. Noteworthy trends emerging from a wealth of studies indicate that generalization is widespread, with a majority of plants associated with multiple pollinator groups and vice versa. Many floral traits, including colour, are continuous rather than discrete variables, making compartmentalization of flowers and pollinators into discrete syndromes problematic. For night-blooming flowers, several studies have reported that those pollinated by moths tend to be white, creamish or yellowish, and that bat-pollinated flowers are creamish in colour, pendulous, have stout pedicels or brush-like stamens [89], although there are several exceptions. In fact, the nocturnal carpenter bee, *Xylocopa tranquebarica* does not appear to show any specific association with floral colours and can collect pollen and nectar from both night-opening pale flowers and day-opening blue, violet or pink flowers as well as from flowers of various shapes and sizes. This nocturnal bee also makes visits by night to flowers that open during the day (and are brightly coloured) in the Asian tropics in Thailand and India [106,107]. The nocturnal Panamanian sweat bee *Megalopta* also visits flowers that are visited by bats [108] and are flower generalists, and have been shown to collect pollen from more than 40 plant species that either had diurnal or nocturnal anthesis [109]. This generalization indicates that floral traits other than colour, such as scent and possibly other cues, are involved in the multimodal signals provided by flowers for nocturnal pollinators. In support of this, two recent studies have shown that neotropical nocturnal sweat bees are attracted to scented baits in Panama [110] and that visits to flowers are limited by light intensity [30]. Hawk moth pollinated flowers tend to be creamish-white or sometimes yellow (although not strictly so), but their visits are also influenced by the flower orientation, shape and corolla curvature [111], as well as by strong scent [33]. Recently, a study of butterfly and moth pollination networks in a rainforest community of 221 plant species on Mount Cameroon concluded that hawk moths were not more specialized than diurnal butterflies, and that they preferred longer, nectar-rich flowers, while being colour generalists [112]. Disentangling the roles of multiple complex traits in the flower choices of nocturnal pollinators is challenging when working with real flowers in natural settings. Therefore, unsurprisingly, behavioural experiments that combine or isolate colour, size and odour in single artificial stimuli have become popular for studying how these traits contribute to foraging choices in diurnal insect pollinators such as honeybees, bumblebees and butterflies [113–115]. These assays have also been used for studying nocturnal hawk moths in the context of foraging [116] and in the context of homing in nocturnal bees [12,117]. This method, though useful, is limited by the inability of artificial stimuli to accurately mimic the complexity of real flowers and pollinator choices. Genetic tools have successively been used to tackle this issue in recent work. For example, using recombinant inbred lines in two species of *Petunia*, Hoballah *et al*. [118] obtained a shift from bee to moth pollination by inducing a change in a single gene that encoded for petal colour.

Thirdly, we know very little about the scale of nocturnal pollination services in tropical and temperate habitats. An analysis across angiosperm families revealed an association between water-holding capacity of plants and night-opening flowers, suggesting that night-blooming is more common in arid habitats as flowering is a water-demanding physiological process [29]. However, data are sparse at the level of plant communities, with most of what we know about the role of nocturnal pollinators coming from studies on one or a few plant species within entire communities. Community-wide pollination network studies tend to be biased towards diurnal pollinators and day-opening flowers, while the structure of nocturnal plant–pollinator networks remains largely unknown. Nocturnal pollination networks such as plant–hawk moth [112] and plant–carpenter bee networks [107] were not found to be more specialized than diurnal networks. However, information on other properties of nocturnal networks is largely unavailable. There is thus clearly a need for further studies to examine these interactions in different tropical and temperate communities to obtain a robust understanding of nocturnal pollination services. An analysis of published studies indicates that there are 227 plant–moth interactions that have been recorded for North America and Europe alone [119] and this number is likely to be much larger for the tropics. The crop potential of nocturnal pollinators also remains largely unassessed [120]. Nocturnal moths including hawk moths, are pollinators of four cucurbit species in Asia [121] and nocturnal bees are pollinators of fruit trees such as cambuci (*Campomanesia phaea*), guaraná (*Paullinia cupana*) and cajá (*Spondias mombin*) in Brazil as well as cucurbits in North America [122]. There is an urgent need for widespread assessment of the status of nocturnal pollinators and their global value as pollinators of wild and crop plants.

### (c) The threat of light pollution

Nocturnal and crepuscular pollinators that use colour vision for finding flowers evolved this ability under the natural illumination provided by the setting sun, the moon and the stars (figure 2). Even though the spectrum of this illumination varies significantly as dusk turns to night, these pollinators have evolved to deal with this change by employing mechanisms of colour constancy (figure 6*d–f*). However, over the last century, anthropogenic sources of light—red-shifted sodium and mercury lamps (figure 2*a*), and more recently, broad-spectrum white LED lighting—have increased in both intensity and geographical spread [123]. What impacts have these new sources of light had on colour vision and pollination services?

Recent studies have revealed that the effect of artificial lighting on pollination services is quite significant [27,124–126]. Experiments that compared pollinator–flower interactions at night on naturally dark meadows with interactions on nearby meadows that were artificially lit, found that flower visitation by nocturnal pollinators was 62% lower on the artificially lit meadow compared to the dark meadow and that this led to a 13% reduction in fruit set in cabbage thistles, even though these flowers were also visited by pollinators during the day [28]. Worse still, these declines had a negative knock-on effect on daytime pollinator communities [28]. Similar conclusions were also made by Macgregor *et al*. [124], who found that moth abundance and species richness around streetlights is dramatically reduced compared to that found over a dark field (by around 50% and 25%, respectively). Moreover, because these moths carry pollen from many different plants (in their study, at least 28 different species), pollen transport (and thus pollination) is also dramatically reduced around street lights (see also [126]).

There are many possible reasons that could explain lower flower visitation rates under artificial lighting. Nocturnal pollinators, such as moths, might simply be lured away from flowers by an artificial light source (reviewed in [127]), or they may even be negatively impacted by some specific physiological reaction to the light source that causes temporal disruption of developmental processes, spatial disorientation or visual disruption [127,128]. Alternatively, changes in the attractiveness of the flowers themselves might also be responsible for visitation decline [129].

Obviously, for a nocturnal pollinator that uses colour vision to find and select flowers, the disturbing unnatural spectra of anthropogenic light sources pose a significant threat [18]. Even the achromatic contrasts of some flowers can be wildly different under artificial lighting than they are in natural nocturnal illumination (figure 7*c*; [18]). In a recent study [130], Briolat and colleagues modelled the impact of various types of artificial illumination on the colour vision of the nocturnal hawk moth *Deilephila* and thus its impacts on *Deilephila's* ability to locate flowers. Interestingly, the impacts depended on the light source. For lamps built using white LEDs, and for mercury vapour lamps, the calculated chromatic contrasts of flowers seen by *Deilephila* against a green foliage background were similar to, or even higher than, contrasts calculated in natural moonlight. However, for lamps built using narrow-band orange LEDs, and for low pressure sodium lamps, *Deilephila's* colour vision completely failed. Chromatic contrasts calculated for Phosphor-Converted (PC) amber LEDs and high-pressure sodium and metal halide lamps varied from natural levels to virtually zero depending on light intensity (i.e. distance from the light source) and flower colour. Even though these results indicate that the impacts of light pollution on colour vision are far from uniform, many commonly used artificial light sources will clearly have a major impact on the ability of nocturnal pollinators to find flowers based on their colour.

## 6. Conclusion

Even though only four species of nocturnal insect pollinators have been confirmed with colour vision, it is highly probable that many more insect species possess this visual ability. Certainly, the presence of at least three opsin classes in most superfamilies of nocturnal moths [15], and the likelihood of three opsin classes in many nocturnal bees, wasps and ants [13], even suggests that nocturnal colour vision may be common within night-active groups of Lepidoptera and Hymenoptera. Having apparently overcome the visual limitations that prevented nearly all nocturnal vertebrates from possessing colour vision, nocturnal insects have evolved the ability to distinguish flowers on the basis of their colour (as well as other sensory cues), thus providing a crucial ecosystem service as pollinators of night-flowering plants. While we are only now starting to realize the vital importance of nocturnal pollination for healthy ecosystem function, it is also becoming apparent that increasing levels of spectrally abnormal anthropogenic light pollution pose a significant threat to the ability of insect pollinators to discriminate colour—and thus flowers—at night. The prevalence of colour vision among nocturnal insect pollinators, as well as its role, together with other senses, in allowing pollinators to distinguish flowers under natural and unnatural illumination, remain open areas for future research.

## Data Availability

This article has no additional data.
